# Efficacy and safety of high-intensity focused ultrasound versus cryoablation for breast fibroadenomas: a systematic review and meta-analysis

**DOI:** 10.3389/fonc.2026.1786278

**Published:** 2026-05-01

**Authors:** YuanFeng Zhao, Jian Sun, Shuai Lin

**Affiliations:** Department of Breast Surgery, Affiliated Hospital of North Sichuan Medical College, Nanchong, China

**Keywords:** breast fibroadenoma, cryoablation, high-intensity focused ultrasound, meta-analysis, minimally invasive therapy

## Abstract

**Methods:**

Up to October 2025, a systematic search was conducted in PubMed, Embase, and Web of Science. Eligible studies included patients with confirmed breast fibroadenoma treated with high-intensity focused ultrasound (HIFU) or cryoablation in prospective or retrospective observational designs, with a sample size >10 and extractable outcome data. The primary outcomes were tumor volume reduction at 6 and 12 months and treatment-related adverse events. In the absence of direct comparative studies, a random-effects meta-analysis of prospective single-arm studies was performed. Study quality was assessed using the Methodological Index for Non-Randomized Studies (MINORS).

**Results:**

Seventeen prospective single-arm studies were included. HIFU achieved pooled tumor volume reduction rates of 54.4% at 6 months and 70.3% at 12 months, with substantial heterogeneity at 12 months (I² = 92.8%). Quantitative pooling for cryoablation was not feasible due to incomplete statistical reporting; descriptive findings suggested greater volume reduction than HIFU. The pooled adverse event rates were 23% for HIFU and 7% for cryoablation (p < 0.05), with significant heterogeneity in both groups (HIFU: I² = 95.6%; cryoablation: I² = 59.2%). No severe complications were reported.

**Conclusion:**

Both HIFU and cryoablation are effective and safe minimally invasive treatments for breast fibroadenomas. Cryoablation may provide superior tumor reduction and fewer adverse events. However, conclusions are limited by the predominance of non-randomized single-arm studies and data heterogeneity. Further high-quality comparative studies are needed.

## Introduction

Fibroadenoma represents the most common benign breast tumor and primarily affects adolescents and young women (1, 2). Epidemiological data suggest that fibroadenomas comprise approximately 68% of all breast masses and account for 44%–94% of breast lesions undergoing biopsy, indicating a large affected population (3). Although most fibroadenomas are asymptomatic and characterized by painless, slow growth, a subset of patients may experience pain, lesion enlargement, or breast deformity (4), with potential negative impacts on quality of life.

Traditionally, surgical excision has been considered the primary treatment option for fibroadenomas that are symptomatic or demonstrate progressive enlargement. However, open surgery may result in breast scarring, morphological changes, and psychological burden, particularly in young women, for whom cosmetic and functional outcomes are of considerable concern (5, 6). With advances in minimally invasive techniques, high-intensity focused ultrasound (HIFU) and cryoablation have emerged as noninvasive or ultra–minimally invasive treatment modalities and have been increasingly applied in the clinical management of breast fibroadenomas (7, 8).

HIFU and cryoablation represent two minimally invasive ablative modalities that have been increasingly applied in the treatment of breast fibroadenomas (9), yet they differ fundamentally in their mechanisms of action and procedural characteristics. HIFU is a noninvasive, extracorporeal technique that delivers focused ultrasound energy to the target lesion, inducing coagulative necrosis and *in situ* tissue destruction while preserving skin integrity (10). In contrast, cryoablation is an image-guided percutaneous procedure that achieves tissue ablation by exposing the lesion to extremely low temperatures (−100 °C or lower) using liquid nitrogen or argon gas, resulting in cellular injury and necrosis (11).

Despite the increasing clinical use of HIFU and cryoablation for breast fibroadenomas, robust comparative evidence on their efficacy and safety remains limited. Existing studies are predominantly small, single-arm cohorts with heterogeneous outcome reporting, limiting evidence-based decision-making. Given the young patient population and the importance of minimally invasive, cosmetically favorable treatments, a quantitative synthesis of available data is clinically necessary. Therefore, this systematic review and meta-analysis was conducted to evaluate and indirectly compare the therapeutic efficacy and safety profiles of HIFU and cryoablation.

## Materials and methods

### Search strategy

Relevant studies published up to October 2025 were identified through a systematic search of PubMed, Embase, and Web of Science. Searches were conducted using combinations of the terms “high-intensity focused ultrasound,” “cryoablation,” and “fibroadenoma.

### Inclusion and exclusion criteria

Studies were eligible for inclusion if they met the following criteria:

1. Involvement of patients with a confirmed diagnosis of breast fibroadenoma;
2. Use of high-intensity focused ultrasound (HIFU) or cryoablation as the treatment modality, including prospective or retrospective observational studies;
3. A total sample size exceeding 10 patients; and
4. Data derived from reliable sources with sufficient and relevant outcome information available for analysis.

Studies were excluded if they:

1. Were conducted in animal models rather than human subjects;
2. Were published as conference abstracts, editorials, discussion papers, or review articles;
3. Did not provide extractable data on treatment outcomes.

Study selection was performed in two stages. First, titles and abstracts were screened for relevance. Full-text articles of potentially eligible studies were subsequently reviewed to determine final inclusion.

### Statistical analysis

All analyses were conducted using Python 3 (NumPy, SciPy, Pandas, and Matplotlib). For single-arm proportional outcomes, including tumor volume reduction rates and adverse event rates, pooled estimates were calculated using the DerSimonian–Laird random-effects model to account for anticipated between-study heterogeneity. Proportions were stabilized with the Freeman–Tukey double arcsine transformation prior to pooling and subsequently back-transformed to obtain overall estimates with corresponding 95% confidence intervals.

### Quality assessment and data extraction

The methodological quality of the included studies was evaluated using the Methodological Index for Non-Randomized Studies (MINORS) (12). As all eligible studies were single-arm, non-randomized in design, assessment was limited to the eight items applicable to non-comparative studies within the MINORS framework. Each item was scored on a scale from 0 to 2, resulting in a maximum possible score of 16. Consistent with commonly adopted thresholds in the literature, studies scoring fewer than 10 points were considered to be at increased risk of bias and were therefore excluded from the final analysis (13).

## Results

### Study selection

**Figure 1:**
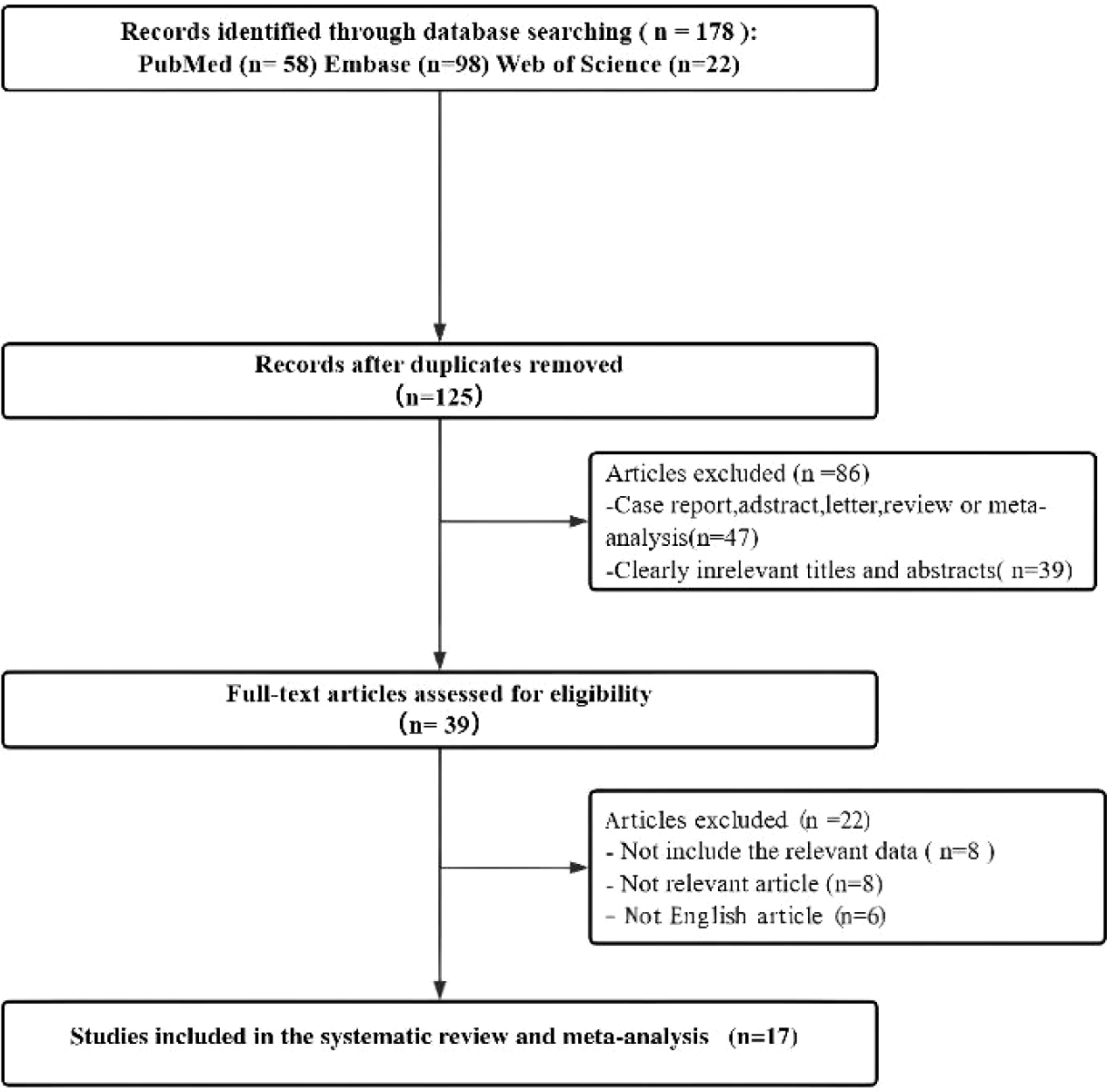
Flow diagram of study selection and exclusion process.

A total of 178 records were initially identified through the literature search. After removal of 53 duplicate articles, 125 records remained for screening. Of these, 47 publications—including case reports, conference abstracts, reviews, and meta-analyses—and 39 records that were clearly irrelevant based on titles and abstracts were excluded. The remaining 39 studies were assessed in full text for eligibility. Subsequently, 8 studies were excluded due to insufficient extractable data, 8 were excluded for irrelevance to the study objectives, and 6 were excluded because they were not published in English. A total of 17 studies were ultimately included. However, due to limitations in data availability and reporting, not all studies were eligible for quantitative synthesis. Studies that did not provide sufficient data for meta-analysis were summarized descriptively and presented in tabular form for reference (14–30). The study selection process is illustrated in the flow diagram (**Figure 1**).

### Assessment of risk of bias

According to the MINORS assessment, the quality scores of the 17 included studies are presented in **Table 1**. All data were collected in accordance with the objectives of the present meta-analysis, and the follow-up duration in each study met the requirements for post-treatment outcome evaluation. All included studies were single-arm in design. The overall MINORS scores of all analyzed studies were ≥12, and no study scored below 10, indicating an overall moderate to high methodological quality.

**Table 1:** MINORS quality scores of the included studies.

Study	Score
Kovatcheva et al.(2015)	15
Peek et al.(2016)	12
Kwong et al.(2021)	14
Yue et al.(2023)	13
Brenin et al.(2019)	14
Boer et al.(2024)	13
Hahn et al.(2018)	14
Peek et al.(2018)	14
Kaufman et al.(2004)	15
Edwards et al.(2005)	13
Kaufman et al.(2005)	14
Kaufman et al.(2004)	15
Durban et al.(2023)	13
Kaufman et al.(2002)	15
Hahn et al.(2012)	13
Plaza et al.(2019)	13
Golatta et al.(2015)	13
Assessed according to the MINORS scale.	

### Study and patient characteristics

**Table 2** summarizes the general characteristics of the included studies and the baseline characteristics of the patients, Seventeen prospective studies were included (8 HIFU and 9 cryoablation), conducted in Europe, Asia, and North America. Sample sizes ranged from 12 to 60 patients, with some studies reporting lesion-based analyses. The mean or median age was generally between 25 and 37 years. Baseline lesion size varied across studies, with HIFU cohorts reporting volumes of approximately 1.0–6.1 cm³ and cryoablation studies generally including lesions around 1–4 cm in diameter. Overall, methodological design was consistent (prospective), but variability existed in sample size and baseline tumor characteristics. **Table 3** presents the treatment modalities and technical characteristics of the HIFU-related studies, including the percentage of lesion volume reduction at 6 and 12 months of follow-up. **Table 4** outlines the treatment and technical characteristics of the cryoablation-related studies and similarly reports the percentage of lesion volume reduction at 6 and 12 months.

**Table 2:** Study and patient characteristics of the included studies.

Author	Year	Study characteristics			Patient characteristics			
		Country	Study design	Surgical method	No. of patients	No. of lesions	Age (years)	Volume of lesions
Kovatcheva et al.	2015	Bulgaria	Prospective	HIFU	42	51	32(16-52)	3.89 (0.34–19.66)cm^3^
Peek et al.	2016	Britain	Prospective	HIFU	20	NA	30.3 ± 7.50	6.1 ± 8.4cm^3^
Kwong et al.	2021	China	Prospective	HIFU	60	NA	37.6(19-60)	2.36 (0.7-2.95) cm
Yue et al.	2023	China	Prospective	HIFU	20	101	25(21.50-31.00)	1.40 (1.10-2.01) cm
Brenin et al.	2019	USA	Prospective	HIFU	20	NA	35.30 ± 10.10	1.80 ± 1.23 (0.57-5.70)cm^3^
Boeer et al.	2024	Germany	Prospective	HIFU	27	NA	28.85± 9.06	1.08 ± 0.80 cm^3^
Hahn et al.	2018	Germany	Prospective	HIFU	27	NA	28.90 ± 9.10	1.08 ± 0.80cm^3^
Peek et al.	2018	Britain	Prospective	HIFU	51	53	29.8 ± 7.2	6.10 ± 12.10 cm^3^
Kaufman et al.	2004	USA	Prospective	Cryoablation	52	53	34 (13–66)	2(0.8-4.2) cm
Edwards et al.	2005	USA	Prospective	Cryoablation	NA	310	NA	NA
Kaufman et al.	2005	USA	Prospective	Cryoablation	32	37	35(13-66)	2.1 ± 0.8 cm
Kaufman et al.	2004	USA	Prospective	Cryoablation	57	70	34 ± 13	2.1 (0.8–4.2) cm
Durban et al.	2023	Spain	Prospective	Cryoablation	12	12	34.7 ± 9.6	4.03 ± 1.35 cm
Kaufman et al.	2002	USA	Prospective	Cryoablation	50	57	NA	2.11 (0.7-4.2) cm
Hahn et al.	2012	Germany	Prospective	Cryoablation	23	23	NA	NA
Plaza et al.	2019	USA	Prospective	Cryoablation	24	26	37.1(19–57)	1.7 ± 0.8 cm
Golatta et al.	2015	Germany	Prospective	Cryoablation	60	60	NA	1.2 ± 0.9 cm

**Table 3:** Technical characteristics of the included HIFU-related studies.

Author	Year	Equipment	Acoustic power(W)	Treatment energy	Treatment time (min)	Volume reduction rate (6 months)	Volume reduction rate (12 months)
Kovatcheva et al.	2015	EchoPulse, Theraclion, Paris, France	NA	NA	118(60-255)	59.2% ± 18.2%	72.5% ± 18.2%
Peek et al.	2016	Theraclion Ltd, Malakoff, France	33.3 ± 4.8	134.6 ± 19.3 J/pulse	34.6 ± 10.5	43.5% ± 38.8%	NA
Kwong et al.	2021	NA	25.00-35.00	NA	NA	NA	62% ± 39.25%
Yue et al.	2023	Chongqing Haifu Medical Technology Co. Ltd., China	149.0 (120.0-189.0)	7.8 (4.4, 18.0) KJ	13.0 (6.0, 23.0)	53.7% ± 13.1%	66.4% ± 10.3%
Brenin et al.	2019	Malakoff, France	38.00 ± 6.10	5.8(1.9-16.3)KJ	43.2(16-81)	51.3% ± 17.3%	68.9% ± 14.7%
Boeer et al.	2024	Theraclion, Malakoff, France	NA	NA	38	62.3% ± 48.08%	86.44% ± 15.45%
Hahn et al.	2018	Theraclion, Malakoff, France	40(40-50)	6.2 ± 2.7 KJ	38.00 ± 12.00	61.6% ± 41.5%	84.8% ± 17.1%
Peek et al.	2018	Theraclion, Malakoff, France	29.1 ± 7.3	117 ± 29.6 J/pulse	32.90 ± 9.80	35% ± 102.8%	43.2% ± 35.4%

NA, not available.

**Table 4:** Technical characteristics of the included cryoablation-related studies.

Author	Year	Equipment	Temperature (°C)	Treatment procedure	Probe diameter(mm)	Operative time(min)	Volume reduction rate (6 months)	Volume reduction rate (12 months)
Kaufman et al.	2004	Sanarus Medical, Pleasanton, California	-186	Freeze–Thaw–Freeze	2.4	NA	69.10%	87.30%
Edwards et al.	2005	Sanarus Medical, Pleasanton, California	NA	NA	2.7	NS	51%	97%
Kaufman et al.	2005	Sanarus Medical, Pleasanton, California	-40	Freeze–Thaw–Freeze	NA	6-30	NA	89%
Kaufman et al.	2004	Sanarus Medical, Pleasanton, California	-160	Freeze–Thaw–Freeze	2.4	14.8 ± 3.3	74%	89%
Durban et al.	2023	Siemens, Berlin, Germany	NA	Freeze–Thaw–Freeze	NA	29.8 ± 11.7	73.95% ± 18%	78.2% ± 13%
Kaufman et al.	2002	Visica Cryoablation System, Sanarus Medical, Pleasanton, California	-160	Freeze–Thaw–Freeze	2.4	6-30	65%	92%
Hahn et al.	2012	Aplio XG, Toshiba, Neuss and IU 22, Philips, Hamburg	NA	Freeze–Thaw–Freeze	3.4	NA	70.10%	75.50%
Plaza et al.	2019	ATEC breast-biopsy system for ultrasound;Hologic;Marlborough, MA	NA	NA	NA	NA	NA	86%
Golatta et al.	2015	IceCure Medical, Israel	-196	Freeze–Thaw–Freeze	3.5	NA	NA	98%

NA, not available.

### Treatment efficacy

**Figure 2:**
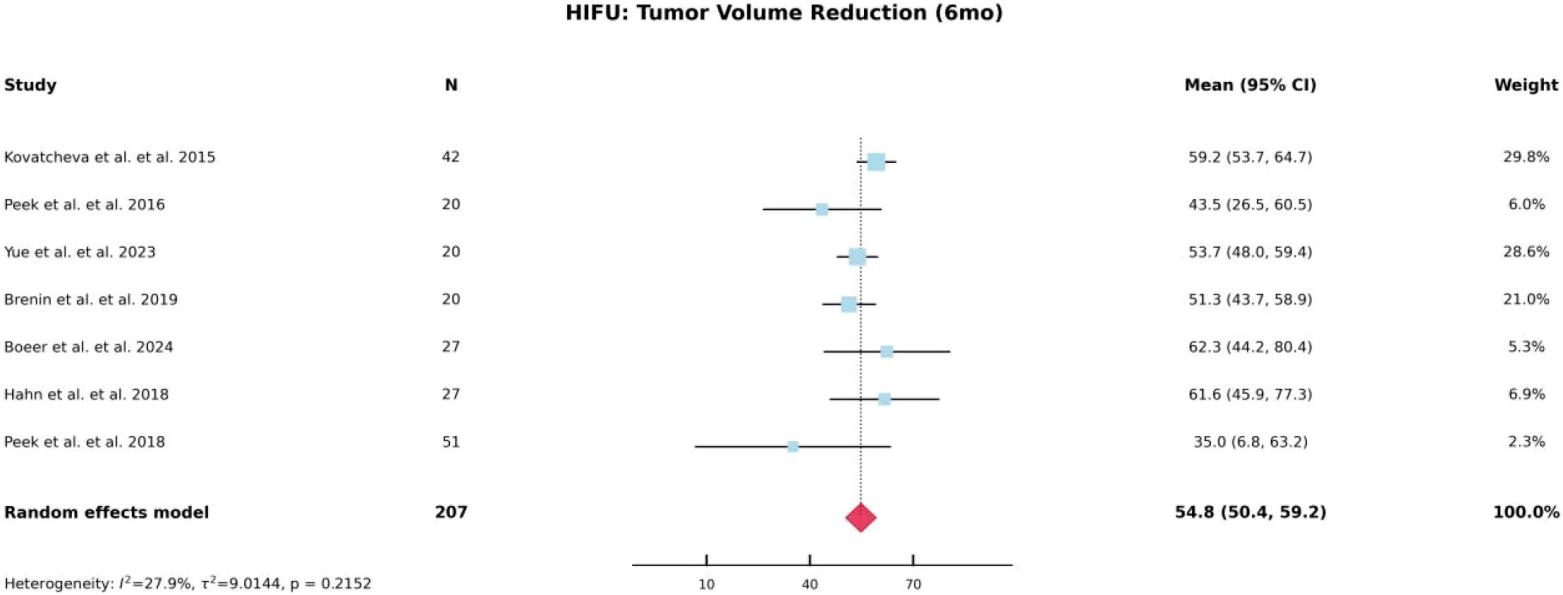
Forest plot of tumor volume reduction rate at 6 months after HIFU.

**Figure 3:**
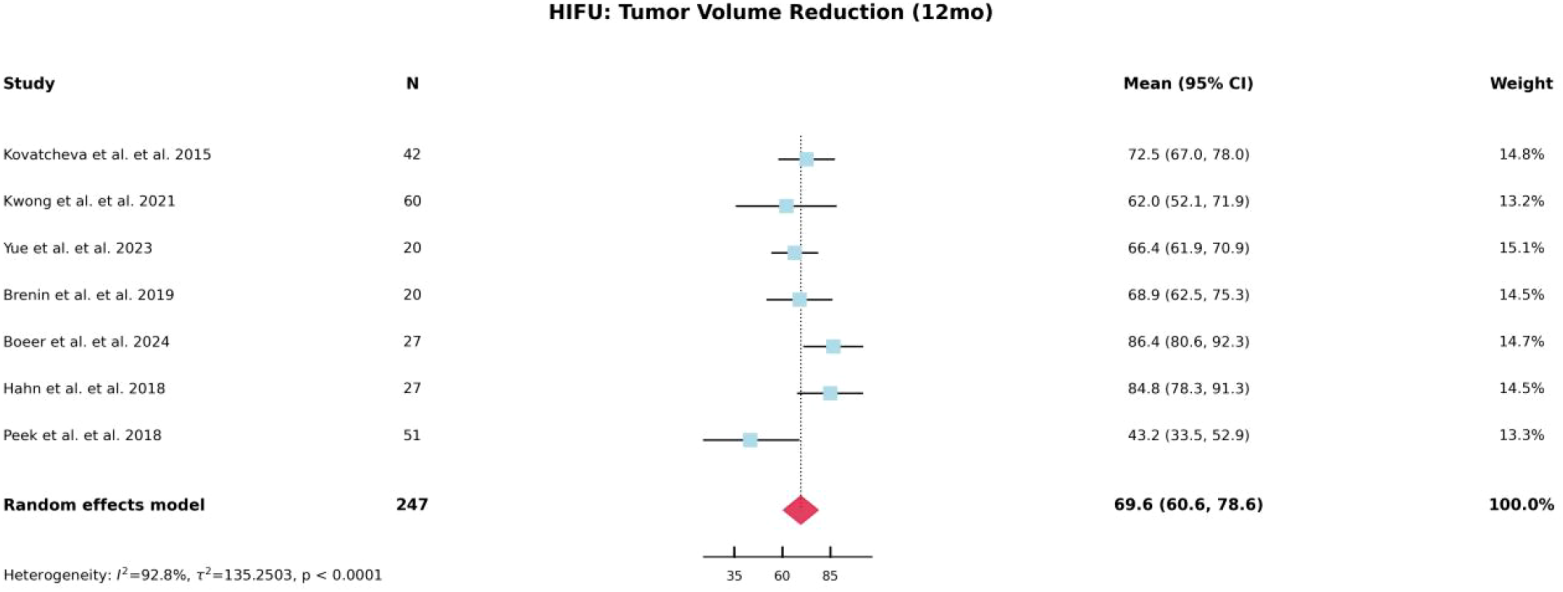
Forest plot of tumor volume reduction rate at 12 months after HIFU.

Based on the characteristics of the included studies, we separately analyzed the percentage of lesion volume reduction at 6 and 12 months following HIFU treatment for breast fibroadenomas. As shown in **Figure 2** and **Figure 3**, the pooled lesion volume reduction rate was 54.4% at 6 months and increased to 70.3% at 12 months, indicating favorable mid- to long-term efficacy of HIFU. In contrast, due to the lack of essential statistical parameters, such as standard deviations, in studies involving cryoablation, quantitative synthesis was not feasible; therefore, the reported volume reduction percentages for cryoablation were descriptively summarized in **Table 4**.

### Incidence of adverse events

**Figure 4:**
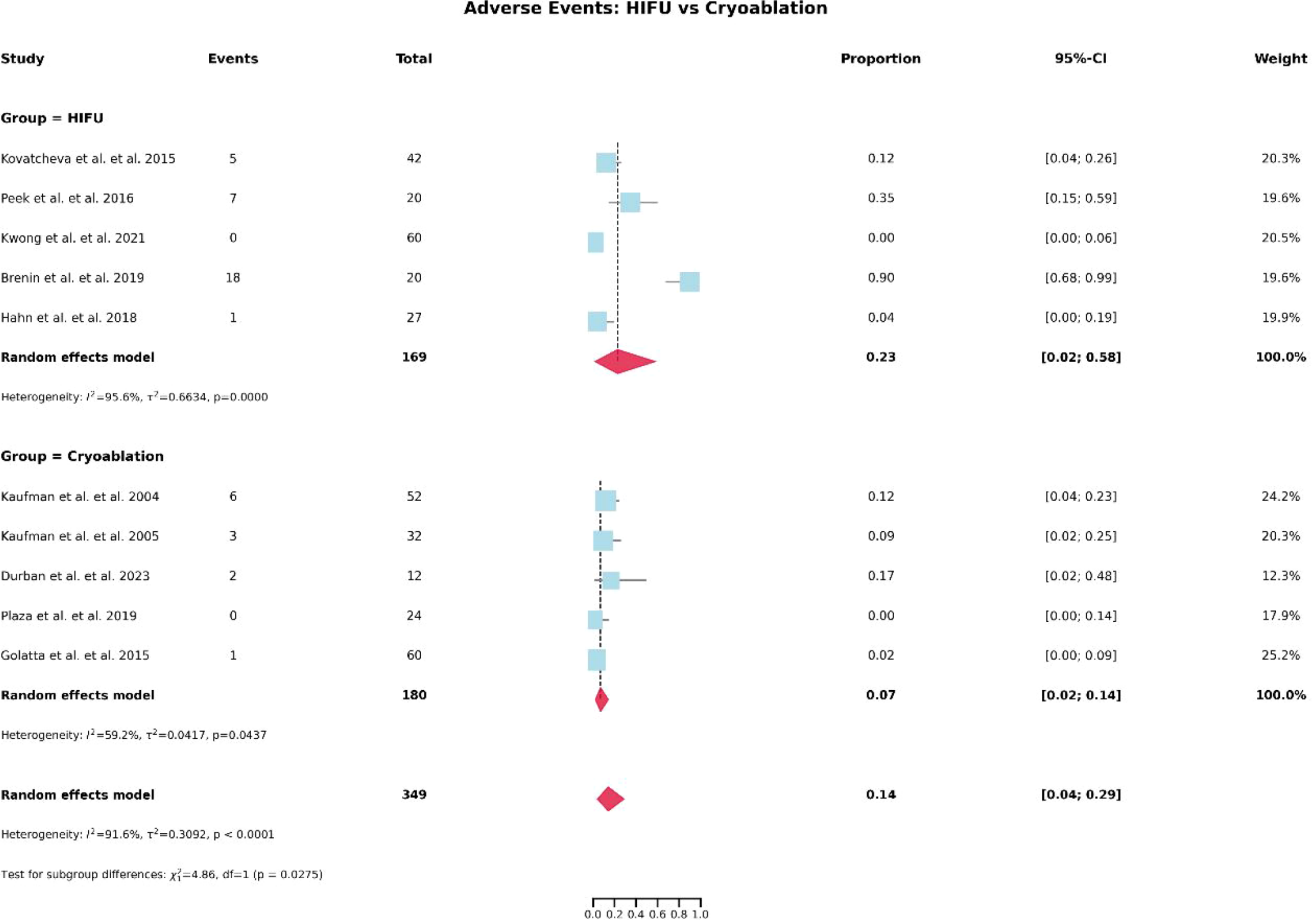
Forest plot of pooled incidence of treatment-related adverse events after HIFU and cryoablation.

**Table 5** summarizes the number of adverse events reported in studies involving HIFU and cryoablation. As illustrated in **Figure 4**, the overall incidence of adverse events was approximately 23% in the HIFU group and 7% in the cryoablation group. Notably, no severe complications were reported in any of the included studies. In HIFU-related studies, the most commonly reported adverse events were mild skin burns, skin hyperpigmentation, and other transient cutaneous reactions. In contrast, the most frequent adverse events associated with cryoablation included postprocedural pain, local skin depigmentation, and scar formation. Overall, the reported complications were predominantly mild to moderate in severity and were generally self-limiting or resolved with conservative management.

**Table 5:** Adverse events after treatment of breast fibroadenomas.

Author	Year	Procedure type	Number of complications	Number of patients	Type of complications
Kovatcheva et al.	2015	HIFU	5	42	Superficial skin burn(n=3) Skin pigmentation(n=1) Palpable subcutaneous induration(n=1)
Peek et al.	2016	HIFU	7	20	Superficial skin burn(n=1) Skin pigmentation(n=6)
Kwong et al.	2021	HIFU	0	60	0
Brenin et al.	2019	HIFU	18	20	NA
Hahn et al.	2018	HIFU	1	27	Pain and fat necrosis(n=1)
Kaufman et al.	2004	Cryoablation	6	52	Local skin depigmentation (n=2) Tape-related blisters (n=2) keloid at probe entry (n=2)
Kaufman et al.	2005	Cryoablation	3	32	Mild tenderness (n=2) Focal pain (n=1)
Durban et al.	2023	Cryoablation	2	12	Treatment-site tenderness (n=2)
Plaza et al.	2019	Cryoablation	0	24	0
Golatta et al.	2015	Cryoablation	1	60	Induration at size of procedure (n=1)

NA, not available.

### Heterogeneity analysis

**Figure 5:**
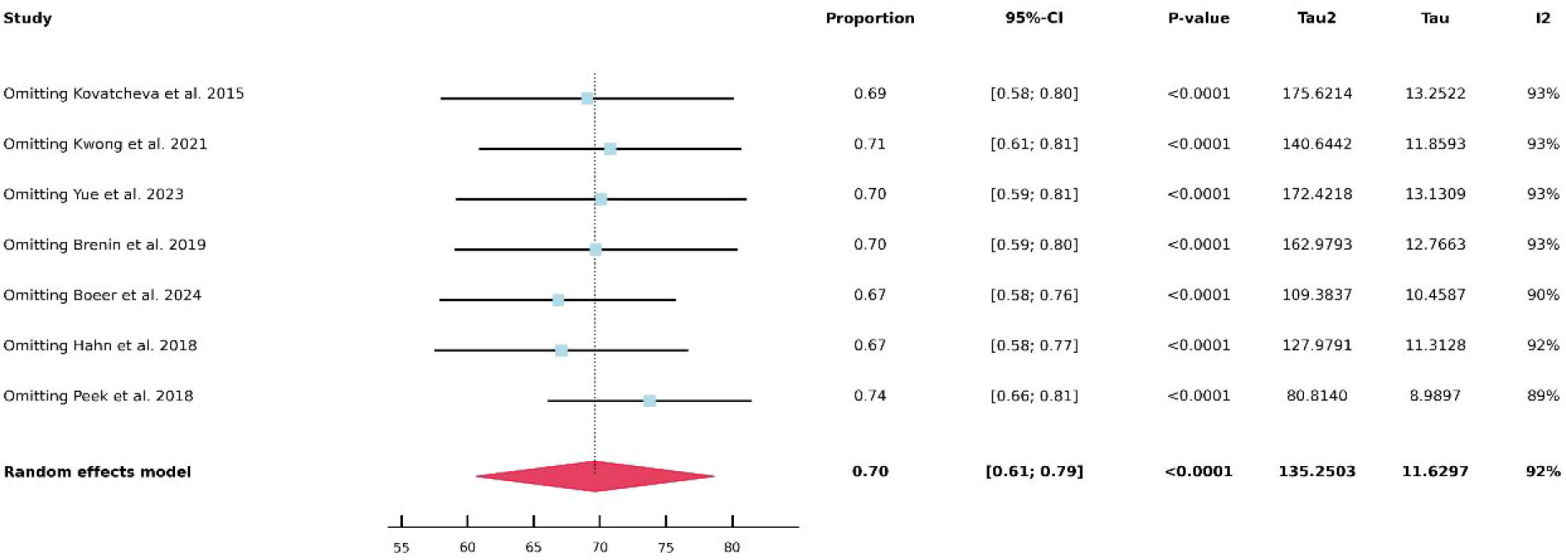
Sensitivity analysis of tumor volume reduction rate at 12 months after HIFU.

**Figure 6:**
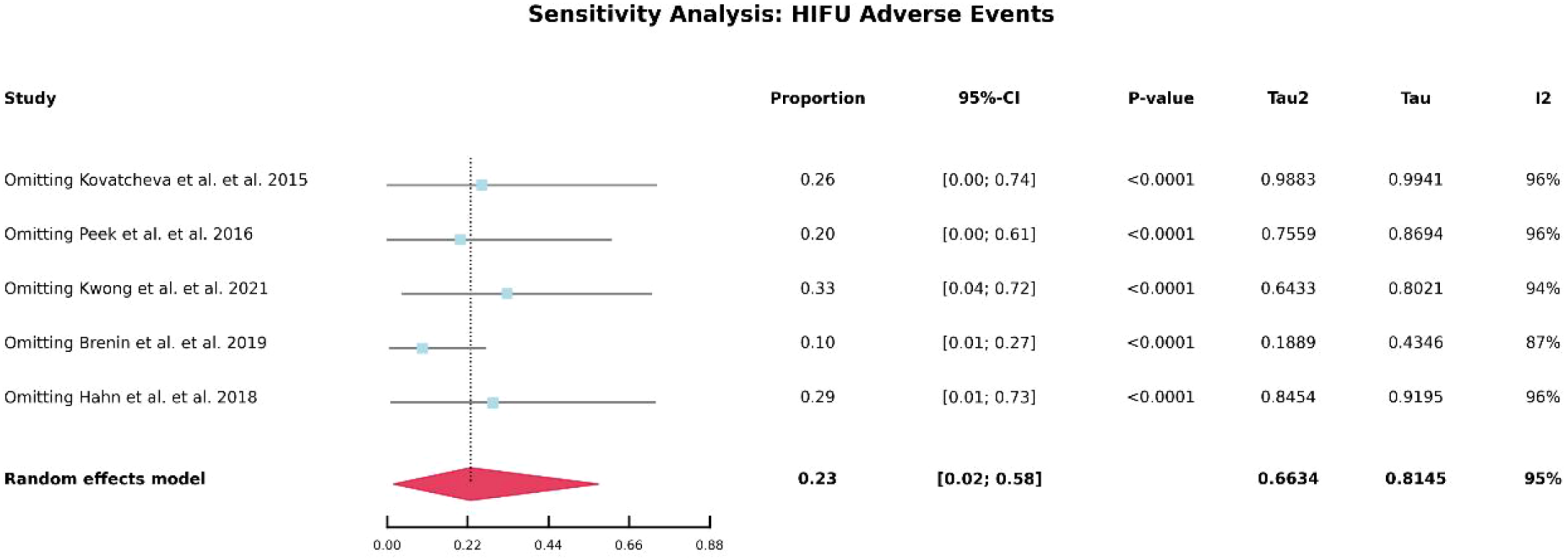
Sensitivity analysis of adverse event incidence after HIFU.

**Figure 7:**
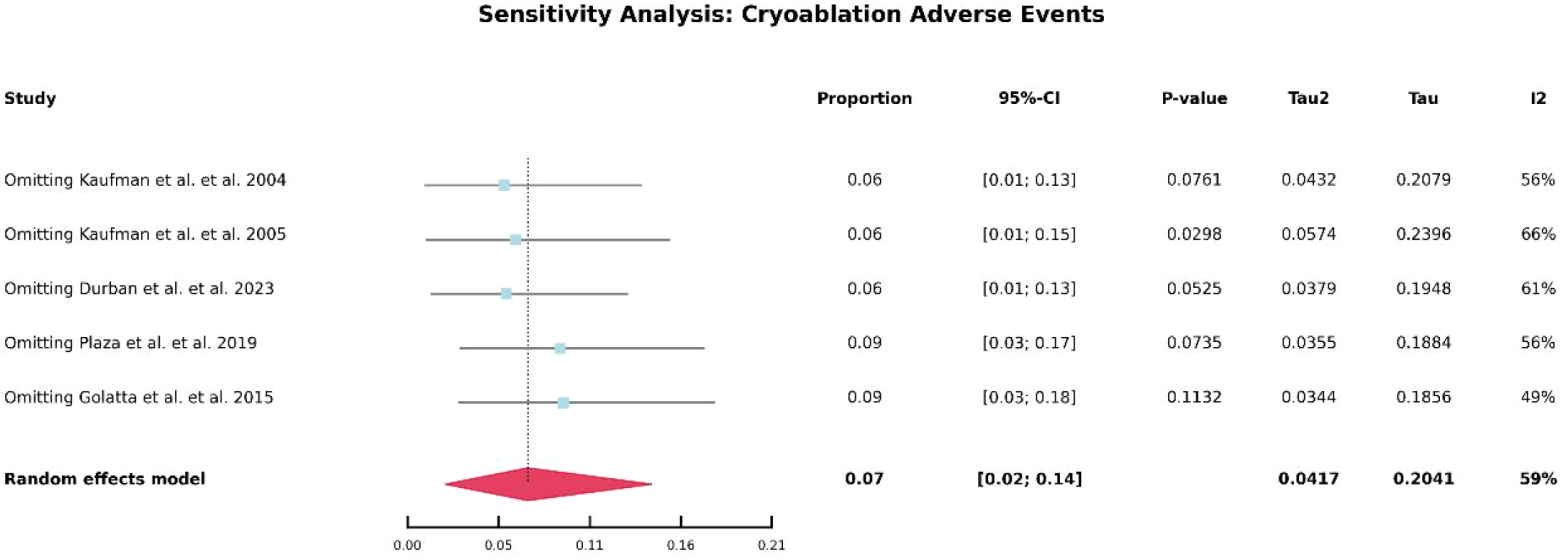
Sensitivity analysis of adverse event incidence after cryoablation.

Due to the substantial heterogeneity observed in the pooled analysis of 12-month lesion volume reduction following HIFU and in the comparison of adverse event rates between HIFU and cryoablation-related studies, further heterogeneity analyses were conducted. As shown in **Figure 5**, no obvious source of heterogeneity was identified in the analysis of 12-month volume reduction after HIFU. Similarly, as illustrated in **Figure 6**, no clear source of heterogeneity was detected among the HIFU studies. In contrast, as shown in **Figure 7**, heterogeneity in the cryoablation studies appeared to be primarily driven by the study conducted by Golatta et al. After exclusion of the Golatta et al. study, the between-study heterogeneity was markedly reduced.

## Discussion

Breast fibroadenomas have a large patient population and predominantly affect adolescents and young women, who generally have high expectations regarding cosmetic outcomes and quality of life. Treatments that result in visible scarring or breast deformity may therefore have a substantial negative impact on both physical and psychological well-being. Consequently, minimally invasive and noninvasive therapeutic approaches have gained increasing attention in recent years. Current evidence suggests that both HIFU and cryoablation demonstrate favorable therapeutic efficacy and high patient satisfaction in the management of breast fibroadenomas. For example, Brenin et al. reported a mean fibroadenoma volume reduction of approximately 68.9% following HIFU treatment, accompanied by a symptom improvement satisfaction rate of 94.4% and a cosmetic satisfaction rate of 100% (18). Likewise, Kaufman et al. demonstrated that cryoablation resulted in a 12-month volume reduction of 87.3%, with an overall patient satisfaction rate of 92% (23). Collectively, these results suggest that both minimally invasive ablation techniques can achieve meaningful tumor reduction while maintaining high levels of patient-reported functional and cosmetic satisfaction.

Although both HIFU and cryoablation have demonstrated favorable clinical outcomes in the treatment of breast fibroadenomas, direct comparative evidence remains unavailable. In this study, HIFU achieved pooled tumor volume reduction rates of 54.4% at 6 months and approximately 70.3% at 12 months. Due to incomplete quantitative data in several cryoablation studies, particularly the absence of standard deviations, a direct pooled comparison was not feasible; however, reported median values suggest that cryoablation may be associated with greater tumor shrinkage. In terms of safety, the incidence of adverse events was higher with HIFU (23%) than with cryoablation (7%), indicating a potentially more favorable safety profile for cryoablation. The observed differences may be attributable to the distinct mechanisms and technical characteristics of the two modalities. Cryoablation induces tumor necrosis through the formation of a low-temperature ice ball, allowing a well-defined and controllable ablation zone under real-time imaging guidance (31, 32), with minimal impact on surrounding tissues and a potential analgesic effect (33). In contrast, HIFU relies on extracorporeal focused ultrasound energy that must traverse the skin and breast tissue, making its efficacy and safety more susceptible to factors such as lesion depth, tissue acoustic properties, and energy distribution, which may increase the risk of thermal skin injury (34, 35). It should be emphasized that the current evidence is largely derived from single-arm studies, and direct comparative data are lacking. Therefore, although cryoablation demonstrated a lower adverse event rate and a trend toward greater tumor volume reduction in this analysis, these findings should be further validated in well-designed prospective comparative studies.

When pooling adverse event rates for HIFU and cryoablation, considerable heterogeneity was observed across the included studies. To further investigate this issue, sensitivity analyses were undertaken. Among studies evaluating HIFU, removal of individual studies did not substantially alter the degree of heterogeneity, and no single study could be identified as a dominant source. This variability may partly reflect differences in how adverse events were defined and reported across studies. For example, pain was the most frequently reported adverse event in the study by Brenin et al., which may be related to the anesthesia or analgesia protocol used in that cohort. Differences in perioperative pain management strategies may therefore have contributed to the observed heterogeneity. In contrast, heterogeneity among cryoablation studies was notably reduced after exclusion of the study by Golatta et al., suggesting that this study accounted for a substantial proportion of the between-study variability in adverse event rates.

Several limitations of the present study should be considered. First, heterogeneity remained evident in the pooled analyses of adverse events for both treatment modalities, particularly for HIFU, despite the use of sensitivity analyses. Such heterogeneity is likely influenced by variations in patient selection, tumor characteristics, perioperative management, and adverse event assessment across studies, which may have affected the robustness and comparability of the pooled estimates. Second, no eligible retrospective studies were identified. The available evidence consisted predominantly of single-arm, non-randomized studies, and no prospective head-to-head comparisons between HIFU and cryoablation were available. Therefore, the pooled estimates were derived from indirect comparisons and should be interpreted with caution, given the potential influence of selection bias and residual confounding. Third, other minimally invasive treatment modalities for benign breast tumors, such as vacuum-assisted excision, were not comprehensively addressed in the present analysis. Vacuum-assisted excision is a widely used alternative to surgical resection and may also serve as a therapeutic option comparable to cryoablation in selected cases. The lack of detailed discussion or comparative evaluation of this modality limits the comprehensiveness of the clinical context in which HIFU and cryoablation were assessed. In addition, incomplete reporting of statistical parameters, such as standard deviations in several cryoablation studies, restricted the feasibility of quantitative pooling for certain outcomes. Accordingly, the findings of this meta-analysis should be interpreted with caution, and further well-designed prospective comparative studies are needed to better define the relative efficacy and safety of HIFU and cryoablation in the treatment of breast fibroadenomas.

Conclusion Overall, the findings of this systematic review and meta-analysis indicate that both HIFU and cryoablation can achieve meaningful reductions in breast fibroadenoma volume, with generally acceptable safety profiles and a low incidence of serious complications. These characteristics support their use as minimally invasive treatment options with favorable cosmetic outcomes. Based on the available data, cryoablation appears to be associated with a trend toward greater tumor volume reduction and fewer reported adverse events. Nevertheless, this observation should be interpreted cautiously, given that most included studies were single-arm and non-randomized, with incomplete reporting of key statistical parameters and a degree of inter-study heterogeneity. Further prospective, well-designed comparative studies with sufficient sample sizes are needed to more definitively determine the relative efficacy and safety of HIFU and cryoablation in the management of breast fibroadenomas.

## Data Availability

The original contributions presented in the study are included in the article/**Supplementary Material**. Further inquiries can be directed to the corresponding author.
