# Supplementary material for: Efficacy and safety of high-intensity focused ultrasound versus cryoablation for breast fibroadenomas: a systematic review and meta-analysis
Source: Front Oncol. 2026 May 1;16:1786278. doi: 10.3389/fonc.2026.1786278 (PMC13175805; doi:10.3389/fonc.2026.1786278)
Supplement: Supplementary file 1 [file Table1.docx]

PubMed检索式

(("Fibroadenoma"[Mesh] OR fibroadenoma*[Title/Abstract]) AND (("High-Intensity Focused Ultrasound Ablation"[Mesh] OR "high intensity focused ultrasound"[Title/Abstract] OR HIFU[Title/Abstract]) OR ("Cryosurgery"[Mesh] OR cryoablation[Title/Abstract] OR cryotherapy[Title/Abstract])))

Embase检索式

('fibroadenoma'/exp OR fibroadenoma*:ti,ab) AND ( ('high intensity focused ultrasound'/exp OR 'high intensity focused ultrasound':ti,ab OR hifu:ti,ab) OR ('cryoablation'/exp OR cryoablation:ti,ab OR cryotherapy:ti,ab))

Web of Science检索式

(("Fibroadenoma"[Mesh] OR fibroadenoma*[Title/Abstract]) AND (("High-Intensity Focused Ultrasound Ablation"[Mesh] OR "high intensity focused ultrasound"[Title/Abstract] OR HIFU[Title/Abstract]) OR ("Cryosurgery"[Mesh] OR cryoablation[Title/Abstract] OR cryotherapy[Title/Abstract])))
